# Evaluation of Wheat Grain and Processing Quality Under Fusarium Head Blight Control Using Strong Oxidizing Radicals

**DOI:** 10.3390/foods14071236

**Published:** 2025-04-01

**Authors:** Huanhuan Zhang, Bo Zhang, Huagang He, Lulu Zhang, Xinkang Hu, Chundu Wu

**Affiliations:** 1 School of Agricultural Engineering, Jiangsu University, Zhenjiang 212013, China; 2111916002@stmail.ujs.edu.cn (H.Z.); zhangll@stmail.ujs.edu.cn (L.Z.); huxk@stmail.ujs.edu.cn (X.H.); 2 School of the Environment and Safety Engineering, Jiangsu University, Zhenjiang 212013, China; 3 School of the Life Sciences, Jiangsu University, Zhenjiang 212013, China; hghe@ujs.edu.cn; 4 Key Laboratory for Theory and Technology of Intelligent Agricultural Machinery and Equipment, Jiangsu University, Zhenjiang 212013, China; 5 Jiangsu Province and Education Ministry Cosponsored Synergistic Innovation Center of Modern Agricultural Equipment, Jiangsu University, Zhenjiang 212013, China

**Keywords:** strong oxidizing radicals, wheat processing, fusarium head blight, quality, green control

## Abstract

Wheat plays a crucial role in global food security; however, in recent years, Fusarium Head Blight (FHB) has severely impacted both wheat yield and quality. Strong oxidative free radicals, with high oxidation potential and rapid reaction rates, offer an effective approach for pollutant degradation and microbial inactivation. In this study, the control effect of strong oxidizing radicals on FHB was evaluated by comparing the untreated control group (JM23), which was infected with FHB, to the experimental group (FG06), which was treated with strong oxidizing radicals following FHB infection. The results show that FG06 achieved a control effectiveness of 87.87%. The study also assessed grain characteristics and milling quality. Statistical analysis revealed that FG06 had a slightly lower flour extraction rate (71.24%) compared to the control wheat (JM23), but it exhibited competitive flour whiteness (81.30) and a gluten index of 85.50%. The dough stability at 10 min was 27.00 FE, while several gelatinization parameters were significantly lower than JM23. However, FG06 had higher protein content (10.94%), flour protein content (10.70%), ash content (0.58%), wet gluten content (28.70%), dry gluten content (9.40%), and sedimentation value (73.00 mL), all significantly higher than those of JM23. Additionally, FG06 had a gelatinization temperature of 68.61 °C, similar to JM23. Overall, Strong oxidizing radicals as an alternative to conventional pesticides not only effectively controls FHB but also maintains or even enhances wheat milling and processing quality, promoting more sustainable agricultural practices.

## 1. Introduction

Wheat (*Triticum aestivum* L.) is one of the most widely cultivated and highest-yielding crops globally [1,2], playing a crucial role in the basic food supply of humanity [3,4]. However, wheat is highly susceptible to various pathogenic microorganisms during production and storage, with Fusarium Head Blight (FHB) caused by *Fusarium* spp. exerting particularly severe effects [5,6]. FHB not only reduces the wheat yield and deteriorates its quality but also produces harmful mycotoxins such as deoxynivalenol (DON) and nivalenol (NIV) [7,8], posing significant threats to human and animal health [9]. Studies have shown that outbreaks of FHB during the wheat heading stage can significantly reduce the grain yield and result in shrivelled kernels, black spots, and varying degrees of quality loss; this ultimately leads to diminished flour quality or even inedibility [10,11,12].

Currently, the primary control methods for FHB include the breeding of resistant varieties [13], the application of chemical fungicides [14,15], and the implementation of field management [12,16]. Although chemical fungicides, particularly triazoles, offer immediate pathogen suppression, their prolonged use raises concerns about pathogen resistance, environmental contamination, and food safety due to residues [17,18]. Therefore, there is an urgent need for a novel technological approach that can effectively inactivate pathogens while minimizing environmental and food-safety risks.

Strong oxidizing radicals (SOR) [19], such as hydroxyl radicals (OH), sulphate radicals (SO_4_^−^), and ozone-derived radicals, have recently garnered attention for their high oxidative potential and rapid reaction rates in applications such as water treatment [20], air disinfection [21], soil remediation, preservation of fresh marine products [22], and agricultural product preservation. These radicals rapidly oxidize and disrupt microbial cell membranes and genetic material [23,24], achieving efficient sterilization [25]. Additionally, they exhibit strong degradation or modification capabilities for certain organic pollutants, including mycotoxins, making them a promising tool for FHB control and toxin management [26,27]. Unlike traditional chemical pesticides applied extensively in fields, strong oxidizing radicals can be generated in situ using specialized equipment (e.g., ozone generators or UV/Fenton reactors), decomposing into environmentally friendly products such as oxygen, water, or sulphate ions; this minimizes secondary pollution. Controlled application can extend their utility to the storage and processing stages [28,29], further enhancing pathogen control [30]. Utilizing ozone micro-nanobubbles (O_3_-MNBs) has been shown to enhance the efficiency of pumpable ice slurry (PIS) production while also extending the shelf life of Mugil cephalus. Experimental results demonstrate that O_3_-MNB injection eliminates supercooling effects during PIS formation, leading to a reduction of more than 7.7% in total freezing time [22]. Moreover, treating tomatoes with a combination of 20/40 kHz ultrasound and an ozone solution (0.85 ± 0.2 mg/L) for 10 min helps maintain and enhance volatile compounds [31]. In addition, pretreating garlic slices with ozone water before drying significantly improved their biochemical properties, resulting in a 7.85% increase in allicin content, a 25.90% rise in total phenolic content, and a 12.31% enhancement in antioxidant activity compared to untreated samples [32].

However, the high reactivity of strong oxidizing radicals may also chemically interact with key nutritional and functional components in wheat grains, such as proteins, starches, and lipids, potentially altering their processing quality. Studies have shown that excessive oxidation can disrupt gluten network structures, weaken dough strength, and oxidize starch granules, affecting dough elasticity and the sensory properties of end products. Furthermore, lipid oxidation may result in off-flavors or reduced nutritional value. Therefore, the successful integration of strong oxidizing radicals into FHB control requires a systematic evaluation of their impact on wheat processing quality to balance pathogen control with quality preservation.

This study introduced strong oxidizing radicals as a green technology for FHB control and evaluated their effects on wheat processing quality. The goal of this research was to contribute to the expansion of green control strategies for FHB while improving the safety and quality of wheat-based products.

## 2. Materials and Methods

### 2.1. Materials

SOR generation system description: Exposure of oxygen molecules to a high-frequency, high-voltage electric field results in ionization and excitation, producing various reactive oxygen species (ROS), including O_2_^+^, O, (O_3_P), O_3_, and O_2_^−^. When dissolved in water, these species undergo further reactions with water molecules to form strong oxidants, such as hydroxyl radicals (•OH), superoxide anions (HO_2_^−^), and ozonide anions (•O_3_^−^), thereby generating a solution rich in strong oxidative radicals. The SOR generation system developed by the research team employs a high-ionization discharge device with a discharge gap of 0.5 mm. The discharge electrode plates are made of sintered silver-coated metal, with ceramic sheets used as the dielectric layer. The gaseous radical generation system has a total power output of 600 W, while the gas–liquid mixing unit operates at 0.75 kW, enabling efficient mixing of gas and liquid. The ozone intake rate is 3.0 L/min, and the system’s pH is precisely controlled at 5.2, ensuring stability and reliability throughout the experiment.

Experimental wheat varieties: “Ji Mai 23”, a wheat variety highly susceptible to ergot disease, bred by the Crop Research Institute of Shandong Academy of Agricultural Sciences, was used as the experimental material for this study.

### 2.2. Field Experiment

The trial was conducted at the Jiangsu Run Guo High-Efficiency Ecological Agriculture Base, located in Zhenjiang, Jiangsu (32.14° N, 119.73° E), using the wheat variety “Ji Mai 23”. The experimental plots were selected from wheat fields within a rice–wheat rotation system that were subjected to natural disease pressure. Each plot covered an area of 20 m^2^, and the experimental treatments were arranged using a completely randomized block design (RCBD), with three replications for each treatment. The field trial employed an advanced real-time spraying device integrated with a synthetic strong oxidant solution. The solution’s working concentration was adjusted to 4.00 mg/L, and the spraying volume was set to 240 L·667 m^2^ (the experimental group was named FG06). In the negative control group, only water was applied (the control group was named JM23). A detailed treatment schedule is provided in Table 1. The timing of the SOR application was determined using a disease warning system in conjunction with meteorological conditions. Applications were made on April 9 (19 °C) and April 16 (22 °C) in 2021 [33].

### 2.3. Control Efficacy Survey

To evaluate pesticide safety, crop growth was observed at 3 and 7 days after application. Field assessments were conducted at 7 and 14 days after the final pesticide treatment to monitor disease incidence and wheat growth. Once Fusarium Head Blight (FHB) symptoms stabilized, the control efficacy was assessed. Two rounds of pesticide application were performed, with five sampling sites selected per test field. Each site was used to record disease incidence in 100 wheat spikes, totaling 500 monitored spikes per treatment group. Disease occurrence was tracked for one month, recording both the total number of spikes and the number of diseased spikes. FHB was monitored from the end of flowering (BBCH 69) to the medium milk stage (BBCH 75). All disease assessment and field efficacy evaluations adhered strictly to the Ministry of Agriculture of the People’s Republic of China (2007) standards (NY/T 1464.15-2007) [34].

$$\mathrm{DI}=\frac{\sum \mathrm{Disease}\;\mathrm{Severity}\;\mathrm{Level}\times \mathrm{Number}\;\mathrm{of}\;\mathrm{Diseased}\;\mathrm{Earsat}\;\mathrm{Each}\;\mathrm{Level}}{\mathrm{Total}\;\mathrm{Number}\;\mathrm{of}\;\mathrm{Ears}\;\mathrm{Surveyed}}\times \frac{100}{7}$$

$$\mathrm{CE}=\frac{\mathrm{Disease}\;\mathrm{Index}\;\mathrm{in}\;\mathrm{the}\;\mathrm{Control}\;\mathrm{Area}-\mathrm{Disease}\;\mathrm{Index}\;\mathrm{in}\;\mathrm{the}\;\mathrm{Treated}\;\mathrm{Area}}{\mathrm{Disese}\;\mathrm{Index}\;\mathrm{in}\;\mathrm{the}\;\mathrm{Control}\;\mathrm{Area}}\times 100$$

### 2.4. Milling and Processing

In order to enhance the toughness of the wheat husk and soften the endosperm structure for a higher extraction rate, we implemented a precise conditioning process prior to grinding the wheat. During this process, the amount of water added was adjusted based on the hardness of the wheat to ensure proper hydration. The conditioning time was set to 24 h. This step ensures uniform moisture content throughout the wheat, preparing it for the subsequent grinding process under optimal conditions.

After conditioning, the wheat was ground using a cyclone sample mill (CT293, FOSS, Hillerød, Denmark) with the following parameters: sample weight of 30 g, speed set to 10,000 rpm, sieve mesh size of 0.8 mm, grinding time of 30 s, and airflow rate maintained at 25 L/min. After grinding, the resulting wheat flour was sieved through an 80-mesh sieve to ensure particle uniformity and remove larger particles, making it suitable for subsequent SDS sedimentation value testing.

For the determination of other indicators, we ground the sample using a laboratory small-scale mill (Quadrumat Junior, Brabender, Denmark). The specific parameters for this process were as follows: sample weight of 30 g, speed set to 3500 rpm, roller gap adjusted to 0.1–0.3 mm, and temperature maintained at room temperature. The ground flour was sieved again through a 60-mesh sieve to remove larger particles and ensure uniformity, thus ensuring the reliability of subsequent testing.

All ground wheat flour samples were sealed in airtight bags and stored at −20 °C prior to use to ensure stability and consistency. The milling yield of wheat milled fractions was calculated using the formula below [35]:

$$\mathrm{Milling}\;\mathrm{yield}\;(\%)=\frac{\mathrm{weight}\;\mathrm{of}\;\mathrm{milling}\;\mathrm{fractions}}{\mathrm{weight}\;\mathrm{of}\;\mathrm{wheat}\;}\times 100$$

### 2.5. Near-Infrared Analysis

The organic components of grains, such as proteins and starches, exhibit distinct absorption spectra in the near-infrared region. Near-infrared spectroscopy (NIRS) has been widely employed for measuring the grain hardness and flour protein content (14% moisture basis). This method is officially recognized by international standard organizations, including the Association of Official Analytical Chemists (AOAC), the American Association of Cereal Chemists (AACC), and the Canadian Grain Commission (NGC).

In this study, an Infratec 1241 Rapid Quality Analyser (FOSS, Hillerød, Denmark) was used to determine the protein and moisture contents of grain samples, as well as the protein and ash contents of wheat flour. Samples were weighed according to the instrument manual. Each sample was tested twice, and the average value of the two measurements was recorded, with retesting conducted for samples with large differences between parallel results.

### 2.6. Flour Color Measurement

The color of the wheat flour was evaluated using a CIELab Color Space System, where L* represents the brightness (ranging from black at 0 to white at 100), a* indicates the green-red axis (negative for green and positive for red), and b* reflects the blue–yellow axis (negative for blue and positive for yellow). Flour whiteness was measured using a CR-410 colorimeter and a WSB-IV intelligent whiteness meter. Each sample was measured three times, with outlier data excluded.

### 2.7. Gluten Content Determination

The wet gluten content, dry gluten content, and gluten index of wheat flour were measured using the ICC155 and AACC38-12 methods with a GM2200 gluten analyzer (Perten Instruments, Huddinge, Sweden). The gluten index, expressed as the percentage of wet gluten retained on the sieve relative to the total wet gluten, was used to assess the gluten strength. Each sample was tested three times, and outliers were removed.

### 2.8. Starch Gelatinization Parameter Analysis

The starch gelatinization properties were evaluated using a Rapid Visco Analyzer (RVA-4, Newport Scientific, Warriewood, Australia) following the AACC22-08 method. For each test, 3.5 g of flour (adjusted to a 14% moisture basis) was mixed with 25 mL of distilled water in an RVA sample canister and homogenized. The viscosity was then measured under controlled temperature and shear conditions using Thermocline for Windows software, version 5.3.2, which automatically processed and plotted the data.

### 2.9. Farinograph Analysis

The dough rheological properties were assessed using a Farinograph-AT (Brabender GmbH & Co. KG, Duisburg, Germany). The test was conducted at a constant room temperature of 22–24 °C, with the mixer bowl temperature maintained at 30 ± 0.2 °C. The water absorption, dough development time, stability, and weakening degree were measured from the farinograph curves. Each sample was tested three times, with outliers excluded.

### 2.10. Data Analysis

All data are presented as the mean ± standard deviation. For the NIRS measurements, each sample was tested twice; for the other measurements, three replicates were performed, and outliers were excluded. Statistical analysis was conducted using SPSS 16.0, with significant differences denoted at *p* ≤ 0.05.

## 3. Results

### 3.1. Disease Control Efficacy

As shown in Table 2,the disease index in the control group was the highest at 28.29, indicating significant disease severity in the untreated wheat field. The disease index in the SOR-treated group dropped to 3.43, considerably lower than in the control, showing strong disease control. An analysis of the efficacy data reveals that the control group, with no treatment, showed no disease control. The SOR-only treatment exhibited an efficacy of 87.87%, demonstrating significant fungicidal effects.

### 3.2. Milling Quality

Flour is the primary raw material for various processed products, and the milling yield, ash content, and flour color are critical parameters for assessing the milling quality. The ash content, which correlates with the mineral content of the grain, affects the flour purity and the sensory quality of processed products. Table 3 shows that the control variety JM23 had an average milling yield of 71.51%, while FG06 exhibited a lower milling yield of 71.24%. FG06 had a slightly higher ash content (0.58%) than JM23 (0.57%).

Flour whiteness is a key determinant of consumer preference. Table 3 shows that the brightness (L*), redness (a*), and yellowness (b*) values of the FG06 flour were 93.19, −0.06, and 6.52, respectively, compared to JM23, which had values of 93.27, −0.14, and 6.94, respectively. Statistical analysis showed significant differences in the brightness and redness of the two samples, while there was no difference in the yellowness. The whiteness values of FG06 and JM23 were 81.40 and 82.00, respectively, with FG06 showing a significant decrease.

### 3.3. Protein Quality

The protein content of wheat grains is highly correlated with both the nutritional and processing quality of wheat. As a crucial storage material in wheat grains, the protein content significantly affects the wheat’s processing qualities. It is also the foundation for gluten formation. Gluten strength, a comprehensive indicator of protein quality, is usually quantified by the gluten index. As shown in Table 4, the protein content of JM23 wheat grains was 12.41%, while for FG06, it was 10.94%. That is, the protein content of FG06 was 11.85% lower than that of the common wheat JM23. Compared with the dry protein content of wheat grains before milling, the protein content of flour milled from YM23 and FG06 has decreased, but the decrease ratio is not the same: YM23 decreased by 5.80%, and FG06 decreased by 2.47%. After wheat milling, there is a significant difference in the flour protein content of YM23 and FG06. According to Table 4, the wet gluten content of JM23 and FG06 was 21.00% and 28.70%, respectively, while the dry gluten content was 7.00 g/100 g for JM23 and 9.40 g/100 g for FG06. This demonstrates that both the wet and dry gluten contents of FG06 were significantly higher than those of JM23. Moreover, compared to JM23, the gluten index of FG06 was decreased by 10.94%. The sedimentation value, as a comprehensive measure of the protein content and quality, also serves as an excellent indicator of protein quality. In this study, the gluten indices of JM23 and FG06 were 96.00 and 85.50, respectively, and the sedimentation values were 52.50 mL and 73.00 mL, respectively.

### 3.4. Starch Gelatinization Properties

Starch is the most abundant component of wheat grains, accounting for approximately 70% of their total composition. The remaining constituents include proteins, moisture, ash, lipids, and other carbohydrates [36]. The quality characteristics of starch significantly influence the processing quality of wheat. Gelatinization properties, which are crucial indicators of starch functionality, directly affect the quality of wheat-based products such as noodles and steamed buns.

As shown in Table 5, all gelatinization parameters, except for the pasting viscosity and gelatinization temperature, showed significant differences. During viscosity measurement, FG06 reached a peak viscosity of 2891 cP at 6.27 min, then decreased to a trough viscosity of 1865 cP, and subsequently increased to a final viscosity of 3146 cP. In contrast, JM23 reached its peak viscosity of 3350 cP at 6.20 min, followed by a drop to a trough viscosity of 2100 cP, and finally a rise to a final viscosity of 3580 cP. Comparison between the two wheat varieties indicated that FG06 had significantly lower peak viscosity, trough viscosity, final viscosity, setback value, and peak time (*p* < 0.01), while no significant differences were found in pasting viscosity and gelatinization temperature.

### 3.5. Farinograph Parameter Analysis

The farinograph results for JM23 and FG06 are summarized in Table 6. Water absorption is influenced by factors such as the moisture content and protein levels of the wheat. In the flour quality tests, JM23 showed a water absorption of 53.50%, while FG06 had a water absorption of 55.70%, a 3.98% increase that was statistically significant. The dough development time is an indicator of both the quantity and quality of gluten. The dough development times for JM23 and FG06 were 1.09 min and 2.18 min, respectively, with a significant difference of 1.09 min. The dough stability time is a measure of resistance to mixing, and FG06 had a dough stability time of 3.59 min, which was 2.16 min higher than JM23, a difference that was highly significant. The degree of softening also reflects the dough’s resistance to overmixing, with JM23 having a softening degree of 90.00 FE at 10 min, while FG06 had a softening degree of 27.00 FE, a significant difference. At 12 min after the peak viscosity, JM23 showed a softening degree of 38.00 FE, while FG06’s was 68.00 FE, showing a highly significant difference of 30 FE. The flour quality index, used to comprehensively evaluate the flour quality, was 110.00 mm for FG06, compared to 21.00 mm for JM23, an increase of 4.24 times.

## 4. Discussion

In the control group JM23, the disease index was 28.29, indicating a high level of disease severity in the absence of treatment, reflecting the natural occurrence of the disease. In contrast, the disease index in the experimental group FG06 dropped to 3.43, with a control effectiveness of 87.87%. This result demonstrates that strong oxidizing radicals are highly effective in controlling FHB, significantly reducing disease occurrence. Therefore, using strong oxidizing radicals as a replacement for conventional pesticides in field control not only achieves comparable control effectiveness but also significantly reduces pesticide use. This, in turn, lowers the environmental impact associated with pesticide application and contributes to the sustainability of wheat production, supporting the transition to more environmentally friendly agricultural practices.

The pursuit of high-quality wheat has been deeply ingrained in agricultural practices. Further research on wheat processing quality can help identify wheat varieties with superior characteristics and provide a foundation for selecting the most appropriate flour for product manufacturing. This study employed various instruments, such as near-infrared grain quality analyzers, rapid viscosity analyzers, automatic farinographs, gluten quantity and quality measuring systems, and colorimeters, to assess five aspects of the FG06 variety, including the seed composition, milling quality, physical properties, protein quality, starch quality, and rheological properties. The 24 parameters that were measured were used to evaluate the quality traits of FG06 and the impact of oxidative free radicals on wheat processing quality under FHB control. Statistical analysis revealed significant or extremely significant differences in 78.26% of the indicators, suggesting that the use of oxidative free radicals may influence the quality traits of common wheat.

Flour is the fundamental raw material for various wheat products, and its milling quality directly influences the product quality. Flour extraction is directly related to the economic benefits of wheat flour enterprises and is one of the main indicators for measuring the quality of wheat flour. The average flour extraction rate of JM 23 was 71.51%, and the average flour extraction rate of FG06 was 71.24%, which was lower than that of the control wheat and there was a significant difference. The flour color and whiteness are important for consumer preferences, especially for products like noodles and steamed buns. Key indicators for evaluating flour color include the whiteness, ash content, and L*, a*, and b* values. The current analysis indicated that the milling quality indicators of FG06 differed from those of JM23. Although FG06 showed a significant decrease in whiteness, it still met market acceptance at a whiteness level of 80, indicating that the whiteness of FG06 is competitive. The flour color is influenced by both genetic and non-genetic factors. Wang and colleagues demonstrated that genotypes primarily determine flour color [37]. Saiz found that ozone can oxidize chromogenic compounds in flour, affecting its color [38].

The quantity and quality of gluten proteins in wheat are closely linked to processing quality. Protein analysis revealed that, compared to JM23, FG06 flour showed significant increases in the protein content, gluten content, and sedimentation value, although the gluten index was significantly decreased. Studies by Huang and others have shown that ozone treatment significantly increases the wet gluten content [39], while Ren noted that ozone alters the gluten protein network structure and enhances elasticity [40]. Research by Zheng and colleagues demonstrated that wheat glutenin and gliadin form a viscoelastic gluten network in dough through their interaction [41]. In the current study, although there was no significant difference in the protein content, the composition of gluten proteins might have changed, affecting the gluten quality. An increase in the gluten content could impact noodle quality by improving the hardness and elasticity of the noodles, while the decreased gluten index suggests that oxidative free radicals weakened the gluten strength, potentially affecting products such as steamed buns and bread, which require high gluten strength.

Starch, the most abundant component in wheat, has significant effects on food processing quality due to its gelatinization characteristics. Starch quality measurements revealed that, although FG06 had a similar gelatinization temperature to JM23, other parameters were much lower. This could be due to changes in the amylose content caused by oxidation. The results demonstrated that after oxidative free radical treatment, most starch gelatinization parameters, including the peak viscosity, were significantly reduced, potentially affecting the quality of noodles and bread. This may be due to the increased amylose content resulting from oxidative free radicals. Additionally, the increased gluten content in this study may contribute to the reduction in the peak viscosity, consistent with findings by Chen [42], where an increase in gluten protein led to a significant reduction in starch gelatinization parameters. The reasons for this include: (1) after oxidation treatment, α-amylase activity in flour is inhibited, reducing starch depolymerization during gelatinization; (2) oxidation of hydroxyl groups to carboxyl groups causes repulsion between negative charges on starch molecules, leading to enhanced water absorption and gelatinization [43].

The farinograph parameters reflect dough rheology and can be used to evaluate gluten strength, which determines flour applications. The farinograph analysis indicated that FG06 exhibited superior dough stability and resistance to mixing compared to JM23. Ozone oxidizes the sulfhydryl groups in gluten proteins, promoting the formation of a cross-linked gluten protein network, thereby enhancing the gluten elasticity and improving the dough water absorption. Li found that ozone treatment significantly increased water absorption and stability, improving the flour properties [44].

FHB infection leads to impaired grain filling, reduced test weight, and a decline in gluten protein and high molecular weight subunits. Additionally, DON remains stable at temperatures below 170 °C and functions as a protein synthesis inhibitor, disrupting metabolic processes in germinating grains [45]. The accumulation of DON is a key factor in FHB-induced deterioration of grain quality. Early suppression of FHB can effectively mitigate disease-related damage to carbon metabolism, thereby promoting subsequent starch accumulation [46]. Furthermore, our previously published data indicate that wheat plants in the treatment groups exhibited significantly higher grain weight per plant, grain number per plant, and thousand-kernel weight compared to the control group [33]. This suggests that, in addition to effectively controlling FHB, strong oxidizing radicals positively influence starch deposition in wheat grains. FHB infection disrupts starch granules, storage proteins, and cell walls, further impacting grain protein composition and quality [47]. The effective application of strong oxidizing radicals reduces FHB severity, thereby limiting toxin accumulation and preserving gluten integrity.

## 5. Conclusions

The results of this study demonstrate that the use of strong oxidizing radicals has a significant effect on the control of Fusarium Head Blight (FHB) in wheat. Specifically, the disease index of FG06 wheat was significantly reduced to 3.43, achieving a control efficacy of 87.87%. In the analysis of processing quality for both JM23 and FG06, the use of strong oxidizing radicals to control FHB led to significant changes in grain composition, resulting in notable alterations in 24 common wheat quality parameters. These changes have profound implications for wheat processing quality, including a significant decrease in starch content and a marked increase in protein content. Regarding milling quality, the use of strong oxidizing radicals significantly or highly significantly reduced flour extraction rate, ash content, and flour whiteness, which may affect both mill production processes and consumer preferences. In terms of protein quality, oxidative free radicals may induce changes in gluten protein composition, leading to a highly significant decrease in gluten index. However, strong oxidizing radical treatment resulted in significant or highly significant positive effects on flour protein content, wet gluten content, dry gluten content, and flour sedimentation value. Regarding starch quality, oxidative free radicals had minimal impact on gelatinization temperature, but they had highly significant negative effects on other gelatinization parameters. Dough rheological characteristics showed significant positive effects in terms of farinograph parameters after the use of strong oxidizing radicals.

The results suggest that strong oxidizing radicals represent a promising avenue for improving wheat quality under FHB conditions. However, additional research is needed to optimize treatment conditions and assess the long-term impacts on both quality and overall plant health. Future studies should focus on examining the potential effects of these treatments on wheat yield and explore ways to refine their application for expanded agricultural use.

## Figures and Tables

**Table 1:** Design of field experiments.

Treatment	JM23	FG06	Application Timing
The first round of application	CK	SOR	BBCH 61
The second round of application	CK	SOR	BBCH 65

Note: BBCH 61: wheat anthesis stage (emergence of the first extruded); BBCH 65: full flowering stage (50% of anthers extruded).

**Table 2:** Statistics of field disease index and prevention efficiency index [33].

Treatment	The Disease Index Under Medication with 14 Days	The Control Effect Under Medication with 14 Days (%)
JM23	28.29	—
FG06	3.43	87.87

**Table 3:** Milling quality of tested wheat grains.

Quality Parameter	JM23 (Mean ± SD)	FG06 (Mean ± SD)	*p*-Value
Milling yield	71.51 ± 0.09	71.24 ± 0.03	0.00
Ash Content (%)	0.57 ± 0.01	0.58 ± 0.00	0.00
L* (%)	93.27 ± 0.06	93.39 ± 0.09	0.00
a* (%)	−0.14 ± 0.04	−0.06 ± 0.02	0.01
b* (%)	6.94 ± 0.03	6.52 ± 0.08	0.36
Flour Whiteness	82.00 ± 0.08	81.30 ± 0.13	0.07

**Table 4:** Protein quality of tested wheat grains.

Quality Parameter	JM23 (Mean ± SD)	FG06 (Mean ± SD)	*p*-Value
Grain Dry Basis Protein (%)	12.41 ± 0.08	10.94 ± 0.07	0.02
Flour Dry Basis Protein (%)	11.69 ± 0.02	10.67 ± 0.03	0.01
Wet Gluten Content (%)	21.00 ± 0.41	28.70 ± 0.46	0.00
Dry Gluten Content (%)	7.00 ± 0.32	9.40 ± 0.00	0.00
Gluten Index (%)	96.00 ± 1.81	85.50 ± 1.92	0.01
Flour Sedimentation Value (mL)	52.50 ± 0.52	73.00 ± 0.77	0.00

**Table 5:** Parameter analysis of starch gelatinization characters.

Quality Parameter	JM23 (Mean ± SD)	FG06 (Mean ± SD)	*p*-Value
Peak Viscosity (cp)	3350.37 ± 11.34	2891.34 ± 8.09	0.00
Trough Viscosity (cp)	2100.00 ± 15.09	1864.69 ± 7.54	0.00
Pasting Viscosity (cp)	1250.57 ± 14.13	1027.67 ± 11.68	0.27
Final Viscosity (cp)	3579.68 ± 23.82	3146.35 ± 5.59	0.00
Setback Viscosity (cp)	1479.67 ± 17.24	1282.27 ± 3.53	0.00
Peak Time (min)	6.20 ± 0.04	6.27 ± 0.00	0.00
Gelatinization Temperature (°C)	67.85 ± 0.00	68.61 ± 0.58	0.23

**Table 6:** Parameter analysis of farinograph.

Quality Parameter	JM23 (Mean ± SD)	FG06 (Mean ± SD)	*p*-Value
Water Absorption (%)	53.50 ± 0.16	55.70 ± 0.21	0.01
Development Time (min)	1.09 ± 0.25	2.18 ± 0.15	0.07
Stability Time (min)	2.16 ± 0.11	3.59 ± 0.06	0.01
10 min Softening Degree (FE)	90.00 ± 7.00	27.00 ± 2.63	0.00
12 min Softening Degree (FE)	38.00 ± 2.63	68.00 ± 4.00	0.00
Flour quality Index (mm)	21.00 ± 4.58	110.00 ± 0.48	0.16

## Data Availability

The original contributions presented in this study are included in the article. Further inquiries can be directed to the corresponding authors.
